# Risk of malignancy after diagnosis of radial sclerosing lesion without atypia

**DOI:** 10.1007/s10549-026-07990-w

**Published:** 2026-06-08

**Authors:** Lorena Beatriz Delgado Casanova, Sagar Dhamne, Erika Resetkova, Mary S. Guirguis, Avanthi Puvvala, Therese B. Bevers, Isabelle Bedrosian, Lei Huo, Kerollos Nashat Wanis

**Affiliations:** 1 Department of Breast Surgical Oncology, The University of Texas MD Anderson Cancer Center, 1400 Pressler Street, Unit 1434, Houston, TX 77030 USA; 2 Universidad Central del Caribe, Bayamón, PR USA; 3 Department of Anatomical Pathology, The University of Texas MD Anderson Cancer Center, Houston, TX USA; 4 Department of Breast Imaging, The University of Texas MD Anderson Cancer Center, Houston, TX USA; 5 Florida Atlantic University, Boca Raton, FL USA; 6 Department of Clinical Cancer Prevention, The University of Texas MD Anderson Cancer Center, Houston, TX USA; 7 Department of Biostatistics, The University of Texas MD Anderson Cancer Center, Houston, TX USA; 8 Department of Health Services Research, The University of Texas MD Anderson Cancer Center, Houston, TX USA; 9 Institute for Data Science in Oncology, The University of Texas MD Anderson Cancer Center, Houston, TX USA

**Keywords:** Radial sclerosing lesion, Complex sclerosing lesion, Radial scar, Benign breast lesions, Imaging surveillance, Surgical excision

## Abstract

**Purpose:**

Surgical excision of radial sclerosing lesions (RSLs) has historically been recommended to rule out malignancy. Contemporary guidelines support imaging surveillance instead whenever these lesions are “adequately sampled.” However, uncertainty exists around what suffices for sampling prior to observation, and the outcomes of imaging surveillance.

**Methods:**

We conducted an observational cohort study including all RSLs diagnosed on image-guided needle biopsy performed at our institution from 2020 to 2025. We excluded lesions with a concurrent diagnosis of invasive cancer, ductal carcinoma in situ, or epithelial atypia in the ipsilateral breast. For lesions that underwent repeat percutaneous biopsy or surgical excision, we estimated the probability that additional sampling revealed malignancy. For the entire population, whether excision was performed or not, we estimated the cumulative incidence of malignancy at the site of RSL during follow-up.

**Results:**

A total of 419 RSLs were diagnosed in 390 patients. 262 (62.5%) lesions were diagnosed by stereotactic-guided vacuum-assisted biopsy (VAB), 100 (23.9%) by MRI-guided VAB, and 57 (13.6%) by ultrasound-guided core needle biopsy. During initial work-up or follow-up, 35 lesions underwent repeat image-guided biopsy and 28 underwent surgical excision. None of these additional sampling procedures revealed malignancy. The estimated 3-year cumulative incidence of malignancy at the RSL site was 0% (95% CI: 0, 2.0%).

**Conclusions:**

These findings support a management strategy of imaging surveillance when radiologic-pathologic concordance is established. Even for less extensively sampled lesions, routine repeat biopsy or surgical excision may not be necessary for RSLs without atypia.

## Introduction

Radial sclerosing lesions (RSLs), including both radial scars and complex sclerosing lesions, are benign breast lesions characterized by a fibroelastotic core and radiating ducts, giving a stellate appearance on imaging [1, 2]. Their diagnosis has become increasingly common due to the widespread adoption of breast cancer screening [3–5]. Despite their benign histology, the imaging features of RSLs often raise concern for an underlying malignancy, and historically these lesions have been managed with surgical excision to ensure complete removal of the radiologic abnormality and to exclude occult cancer [1, 6–8].

In recent years, the management of RSLs diagnosed without associated epithelial atypia has shifted towards radiologic surveillance [9–12]. Current clinical practice guidelines support observation of these lesions whenever they have been “adequately sampled,” [13, 14] in part based on evidence that RSLs diagnosed by vacuum-assisted biopsy (VAB) are unlikely to harbor malignancy [15]. Because many patients undergo initial biopsy without this degree of sampling, these recommendations lead to repeat biopsies and excisions in many cases [16–19].

A recent meta-analysis, including 49 studies, reported that the upgrade rate to malignancy is 1% for RSL without associated atypia diagnosed by VAB, compared with 5% for those diagnosed by 14-gauge core needle biopsy [15]. However, because outcomes for patients who underwent observation are not typically reported, and observation is increasingly common, these upgrade rates may overestimate the true risk of concurrent malignancy. Patients selected for observation may have lower-risk radiologic features, and their outcomes are not captured in surgical cohorts.

To address these limitations of the available literature, we designed this study to evaluate the outcomes of RSLs diagnosed on image-guided biopsy, irrespective of whether they underwent excision or active monitoring. Using data from a cohort of patients diagnosed with RSL on image-guided biopsy, we estimated the risk of upgrade to malignancy when repeat biopsy is undertaken for “adequate sampling,” the risk of upgrade at surgical excision, if performed, and the overall risk of malignancy during follow-up at the RSL site regardless of whether surgical excision or imaging surveillance was performed. Our aim was to clarify the diagnostic yield of additional sampling after initial biopsy during RSL work-up, and the safety of imaging surveillance for RSLs.

## Methods

We used institutional electronic health record data to identify all diagnoses of RSL (radial sclerosing lesion, radial scar, or complex sclerosing lesion) by image-guided biopsy (including VAB) performed at The University of Texas MD Anderson Cancer Center between July 2020 and May 2025. The core biopsy pathology for each RSL was reviewed by an independent pathologist (LH, SD, or ER) to verify the histologic diagnosis. We excluded cases in which patients had a concurrent diagnosis of invasive cancer, in situ carcinoma, or atypia (lobular carcinoma in situ, atypical ductal hyperplasia, atypical lobular hyperplasia, flat epithelial atypia, atypical papillary lesion, or atypical apocrine adenosis) in the ipsilateral breast, whether at the site of the RSL or elsewhere in the ipsilateral breast. The University of Texas MD Anderson Cancer Center institutional review board approved the study.

### Variables

Clinical, radiologic, and pathologic data were collected for the cohort. We collected data on patient demographics and risk factors for malignancy including family history of breast cancer, the presence of a pathogenic variant predisposing to malignancy, prior breast surgery or biopsy, and the presence of malignancy or atypia (atypical ductal hyperplasia [ADH], atypical lobular hyperplasia [ALH], lobular carcinoma in situ [LCIS], or other types of atypia including flat epithelial atypia, atypical papillary lesions, and atypical apocrine adenosis) in the contralateral breast at the time of RSL diagnosis. Lesion-specific characteristics were recorded including the imaging modalities used for diagnosis of the lesion (mammography, ultrasound, and/or MRI), and the lesion size and appearance on each modality. Biopsy-specific data included biopsy technique, categorized as MRI-guided, Stereotactic-guided VAB, and ultrasound-guided; needle gauge size; and number of cores extracted. Pathology results were recorded for the initial biopsy, any subsequent biopsies performed, and for surgical excision if undertaken during the initial work-up or in follow-up. For patients who did not undergo surgical excision, any subsequent ductal carcinoma in situ or invasive carcinoma diagnosis at the site of the RSL during radiologic surveillance was recorded.

### Statistical analysis

We described the distribution of demographic and breast cancer risk factor characteristics for the entire cohort. We summarized the work-up performed for each RSL including the diagnostic imaging performed and the identification of any concurrent contralateral breast lesions, stratified by the imaging modality used to guide the biopsy. Likewise, we described the lesion characteristics and biopsy details (needle gauge size and number of sampled cores) stratified by the imaging modality used to guide the biopsy.

We reported the proportion of lesions for which a repeat biopsy was performed for additional sampling of the RSL during initial work-up, and the probability of upgrade to invasive carcinoma, ductal carcinoma in situ (DCIS), or a lesion with epithelial atypia. Similarly, we reported the proportion of lesions for which surgical excision was performed during initial work-up and the probability of upgrade on final surgical pathology.

Most patients in this study did not undergo surgical excision. Thus, for the entire cohort, we estimated the 1-, 3-, and 5-year cumulative incidence of upgrade to DCIS or invasive carcinoma at the site of the RSL under yearly imaging follow-up. Upgrades could occur during initial work-up, including repeat biopsy or surgical excision, or during imaging surveillance where a change in lesion appearance prompted repeat biopsy or surgical excision. To estimate outcomes under continuous surveillance, observations were censored at the date of their expected annual imaging if they exceeded a yearly interval without breast imaging. To estimate cumulative incidences, we used the Kaplan-Meier estimator with non-parametric confidence intervals (beta product confidence procedure) due to few expected outcome events [20, 21]. The planned analysis included stratification by risk factors, including baseline patient risk factors and lesion characteristics, but these analyses were not performed because no malignancy events were observed during data collection. Analyses were performed using R version 4.4.3.

## Results

### Lesion and patient characteristics

Over the study period, a total of 419 RSLs were diagnosed on image-guided biopsy in 390 patients. The patient characteristics for all RSL diagnoses are displayed in Table 1. The median patient age at RSL diagnosis was 54.1 years (interquartile range: 44.8, 64.8 years). There was a reported family history of breast cancer in 226 (53.9%) cases and a known breast-cancer associated genetic pathogenic variant in 24 (5.7%) cases.

**Table 1:** Patient characteristics for radial sclerosing lesion diagnoses

Variable	level	Overall
No.		419
Age at diagnosis [interquartile range]		54.1 [44.8, 64.8]
Patient race	American Indian or Alaska Native	4 (1.0)
	Asian	31 (7.4)
	Black	38 (9.1)
	White	295 (70.4)
	Other or unknown	51 (12.2)
Patient ethnic group	Hispanic or Latino	81 (19.3)
	Not Hispanic or Latino	324 (77.3)
	Unknown	14 (3.3)
Family history of breast cancer	Yes	226 (53.9)
	No	193 (46.1)
Pathogenic genetic variant^a^	Yes	24 (5.7)
	No	395 (94.3)
Prior history of any breast surgery	Yes	85 (20.3)
	No	334 (79.7)

Numbers are No. (%), unless otherwise stated. ^a^Pathogenic variants considered to be strongly associated with breast cancer included: ATM, BARD1, BRCA1, BRCA2, CDH1, CHEK2, NF1, PALB2, PTEN, RAD51C, RAD51D, STK11, TP53

Prior to evaluation and biopsy at our institution, 40 (9.5%) lesions had undergone work-up and biopsy elsewhere. During work-up at our institution, prior to biopsy, imaging included diagnostic mammography in 386 (92.1%) cases, ultrasound in 381 (90.9%), and MRI in 146 (34.8%). Most lesions (262 [62.5%]) underwent initial biopsy with stereotactic-guided VAB, while 100 (23.9%) underwent MRI-guided VAB, and 57 (13.6%) underwent ultrasound guided core needle biopsy. Most patients did not have concurrent contralateral breast atypia or malignancy at the time of RSL diagnosis. Table 2 details the diagnostic work-up and presence of contralateral lesions stratified by initial biopsy modality.

**Table 2:** Radial sclerosing lesion work-up and concurrent pathologies by initial biopsy modality

Variable	level	Ultrasound guided core needle biopsy	Stereotactic guided VAB	MRI guided VAB
No.		57	262	100
Diagnostic mammogram performed^a^	Yes	54 (94.7)	262 (100.0)	70 (70.0)
	No	3 (5.3)	0 (0.0)	30 (30.0)
Breast ultrasound performed^a^	Yes	57 (100.0)	252 (96.2)	72 (72.0)
	No	0 (0.0)	10 (3.8)	28 (28.0)
Breast MRI performed^a^	Yes	12 (21.1)	34 (13.0)	100 (100.0)
	No	45 (78.9)	228 (87.0)	0 (0.0)
Concurrent contralateral atypical ductal hyperplasia	Yes	1 (1.8)	6 (2.3)	8 (8.0)
	No	56 (98.2)	256 (97.7)	92 (92.0)
Concurrent contralateral atypical lobular hyperplasia	Yes	1 (1.8)	2 (0.8)	6 (6.0)
	No	56 (98.2)	260 (99.2)	94 (94.0)
Concurrent contralateral lobular carcinoma in situ	Yes	1 (1.8)	2 (0.8)	7 (7.0)
	No	56 (98.2)	260 (99.2)	93 (93.0)
Concurrent contralateral other atypia^b^	Yes	1 (1.8)	2 (0.8)	2 (2.0)
	No	56 (98.2)	260 (99.2)	98 (98.0)
Concurrent contralateral ductal carcinoma in situ	Yes	0 (0.0)	13 (5.0)	18 (18.0)
	No	57 (100.0)	249 (95.0)	82 (82.0)
Concurrent contralateral invasive cancer	Yes	4 (7.0)	18 (6.9)	14 (14.0)
	No	53 (93.0)	244 (93.1)	86 (86.0)

Numbers are No. (%). ^a^Imaging modalities performed during the work-up period immediately prior to biopsy. ^b^Other atypia included flat epithelial atypia, atypical papillary lesions, and atypical apocrine adenosis

Among the 262 RSLs diagnosed by stereotactic-guided VAB, the corresponding lesion on mammography was an architectural distortion in 180 (68.7%) lesions, calcifications in 54 (20.6%), focal asymmetry in 21 (8.0%), and mass in 6 (2.3%). All stereotactic-guided VABs were performed using a 9-gauge needle and most (225 [85.9%]) sampled 12 or more biopsy cores. Of the 57 RSLs diagnosed by ultrasound-guided core needle biopsy, the corresponding lesion on ultrasound was a mass in 48 (84.2%) lesions, an architectural distortion in 4 (7.0%), and a hypoechoic area in 3 (5.3%). The majority (51 [89.5%]) of ultrasound-guided core needle biopsies were performed using a 14-gauge needle, with most (46 [80.7%]) sampling fewer than 6 cores. Of the 100 RSLs diagnosed by MRI-guided VAB, the corresponding lesion on MRI was non-mass enhancement in 63 (63.0%) lesions, a mass in 34 (34.0%), and architectural distortion in 3 (3.0%). All MRI-guided VABs were performed using a 9-gauge needle and most (92 [92.0%]) sampled 12 or more biopsy cores. Lesion characteristics and biopsy details, stratified by initial biopsy modality, are displayed in Table 3.

**Table 3:** Biopsy details for radial sclerosing lesions by initial biopsy modality

Variable	level	Ultrasound guided core needle biopsy	Stereotactic guided VAB	MRI guided VAB
No.		57	262	100
Lesion appearance on imaging	Calcifications	0 (0.0)	54 (20.6)	0 (0.0)
	Mass	48 (84.2)	6 (2.3)	34 (34.0)
	Hypoechoic area	3 (5.3)	0 (0.0)	0 (0.0)
	Architectural distortion	4 (7.0)	180 (68.7)	3 (3.0)
	Focal asymmetry	0 (0.0)	21 (8.0)	0 (0.0)
	Non-mass enhancement	0 (0.0)	0 (0.0)	63 (63.0)
	Other	2 (3.5)	1 (0.4)	0 (0.0)
Abnormality size	< 1 cm	32 (56.1)	63 (24.0)	38 (38.0)
	1–2 cm	18 (31.6)	98 (37.4)	33 (33.0)
	> 2 cm	7 (12.3)	76 (29.0)	24 (24.0)
	Not specified	0 (0.0)	25 (9.5)	5 (5.0)
Biopsy needle gauge	9 G	0 (0.0)	262 (100.0)	100 (100.0)
	12 G	1 (1.8)	0 (0.0)	0 (0.0)
	14 G	51 (89.5)	0 (0.0)	0 (0.0)
	16 G	3 (5.3)	0 (0.0)	0 (0.0)
	21 G	2 (3.5)	0 (0.0)	0 (0.0)
Number of biopsy cores	< 6	46 (80.7)	0 (0.0)	0 (0.0)
	6–11	11 (19.3)	37 (14.1)	8 (8.0)
	≥ 12	0 (0.0)	225 (85.9)	92 (92.0)

Numbers are No. (%)

### Repeat biopsy and surgical excision

During the initial work-up, repeat image-guided biopsy was performed in 30 of 57 RSLs (52.6%) diagnosed via ultrasound-guided biopsy, 2 of 262 RSLs (0.8%) diagnosed via stereotactic-guided VAB, and 0 of 100 RSLs (0%) diagnosed via MRI-guided VAB. All repeat biopsies were VABs performed with a 9-gauge needle and 12 or more sampled biopsy cores.

In the 30 RSLs initially diagnosed by ultrasound-guided biopsy that underwent repeat biopsy by VAB for additional sampling, 0 of 30 (0%) had invasive cancer or DCIS on repeat biopsy, while 3 of 30 (10%) had atypia diagnosed on repeat biopsy. In the 2 RSLs initially diagnosed by stereotactic-guided biopsy that underwent repeat VAB, 0 of 2 (0%) had invasive cancer or DCIS on repeat biopsy, while 1 of 2 (50%) had atypia.

In total, 3 RSLs initially diagnosed by ultrasound-guided biopsy underwent surgical excision during the initial work-up. Of these, 0 (0%) had invasive cancer or DCIS on surgical pathology, and 0 (0%) had atypia. Similarly, 5 RSLs initially diagnosed by stereotactic-guided VAB underwent surgical excision during the initial work-up and 0 (0%) had invasive cancer or DCIS on surgical pathology, while 2 (40.0%) had atypia. Lastly, 11 RSLs initially diagnosed by MRI-guided VAB underwent surgical excision during the initial work-up and 0 (0%) had invasive cancer or DCIS on surgical pathology, while 3 (27.3%) had atypia. Of the 19 lesions that underwent surgical excision during initial work-up, 12 (63.2%) did so because of patient preference, 1 (5.3%) because of atypia identified on repeat biopsy performed for additional sampling, 5 (26.3%) because of multidisciplinary assessment of discordance or recommendation for additional sampling, and 1 (5.3%) was excised incidentally during reduction mammoplasty.

### Risk of malignancy during follow-up

**Fig. 1:**
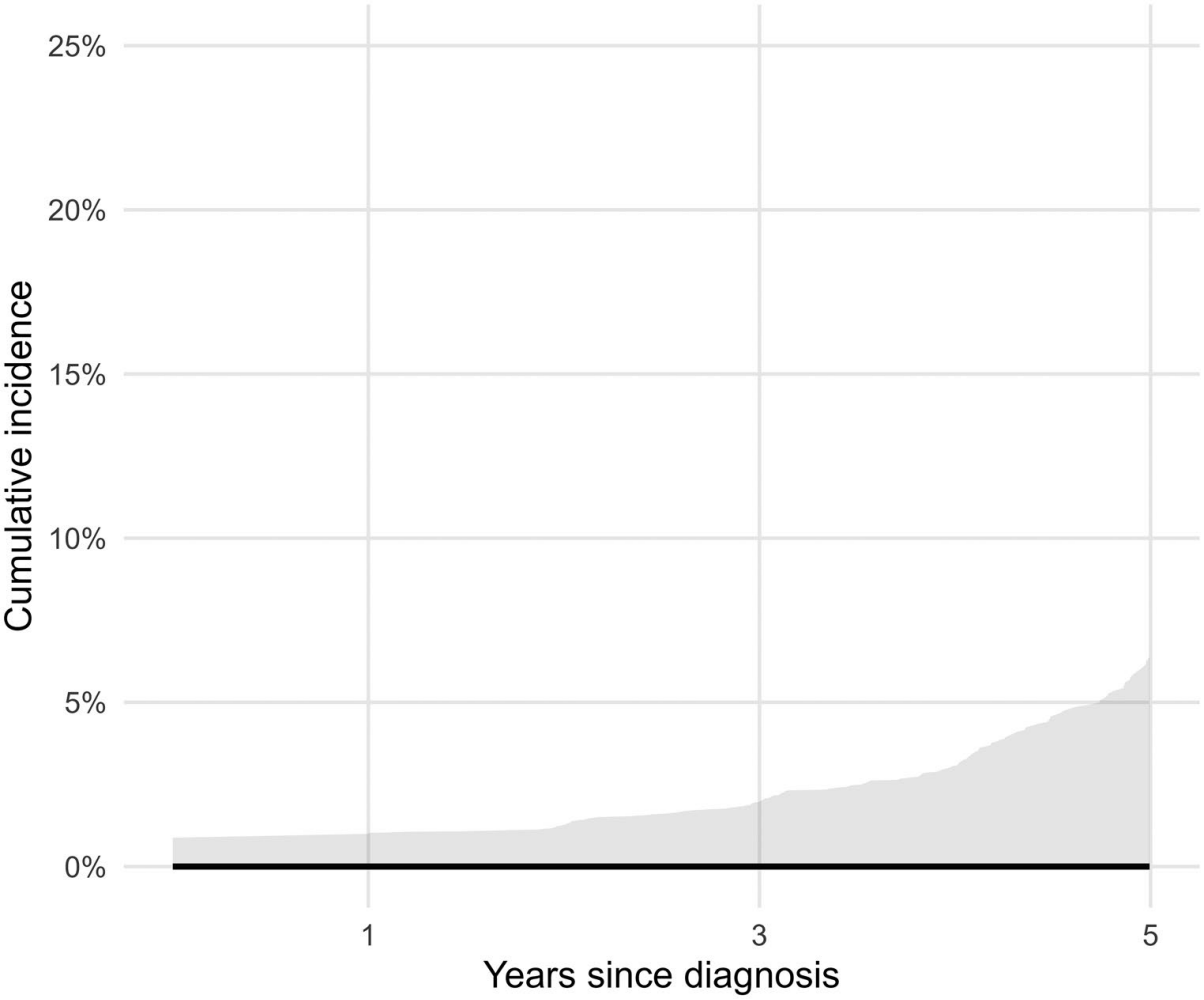
Cumulative incidence of DCIS or invasive cancer at the site of the RSL under annual breast imaging surveillance. Shaded area represents 95% confidence interval

Median follow-up with annual imaging was 2.7 years (interquartile range: 1.9, 4.1 years). Follow-up was at least 2 years for 278 lesions (66.3%) and at least 5 years for 55 lesions (13.1%). During annual surveillance, 3 patients underwent repeat image-guided biopsy by VAB due to a change in the lesion appearance, and 9 underwent surgical excision. None of these biopsies or excisions revealed invasive carcinoma or DCIS. Therefore, the cumulative incidence of malignancy at the RSL site under annual imaging surveillance was 0% (95% CI: 0, 1.0%) at 1-year, 0% (95% CI: 0, 2.0%) at 3-years, and 0% (95% CI: 0, 6.4%) at 5-years following diagnosis. The cumulative incidence curve and corresponding 95% confidence interval is displayed in Fig. 1. Details of imaging-guided biopsies and surgical excisions with their respective pathologies are displayed in Table 4.

**Table 4:** Outcomes of subsequent image-guided biopsy or surgical excision, stratified by initial biopsy modality

		Invasive cancer	Ductal carcinoma in situ	Atypia
***Initial stereotactic-guided VAB (*** ***n*** *** = 262)***				
** Additional sampling during initial work-up**				
Repeat imaging-guided biopsy		0 of 2 (0.0%)	0 of 2 (0.0%)	1 of 2 (50.0%)
Surgical excision		0 of 5 (0.0%)	0 of 5 (0.0%)	2 of 5 (40.0%)
** Procedures performed during follow-up**				
Imaging-guided biopsy		0	0	0
Surgical excision		0 of 2 (0.0%)	0 of 2 (0.0%)	0 of 2 (0.0%)
***Initial MRI-guided VAB (*** ***n*** *** = 100)***				
** Additional sampling during initial work-up**				
Repeat imaging-guided biopsy		0	0	0
Surgical excision		0 of 11 (0.0%)	0 of 11 (0.0%)	3 of 11 (27.3%)
** Procedures performed during follow-up**				
Imaging-guided biopsy		0	0	0
Surgical excision		0 of 5 (0.0%)	0 of 5 (0.0%)	0 of 5 (0.0%)
***Initial ultrasound-guided core needle biopsy (*** ***n*** *** = 57)***				
** Additional sampling during initial work-up**				
Repeat imaging-guided biopsy		0 of 30 (0.0%)	0 of 30 (0.0%)	3 of 30 (10.0%)
Surgical excision		0 of 3 (0.0%)	0 of 3 (0.0%)	0 of 3 (0.0%)
** Procedures performed during follow-up**				
Imaging-guided biopsy		0 of 3 (0.0%)	0 of 3 (0.0%)	0 of 3 (0.0%)
Surgical excision		0 of 2 (0.0%)	0 of 2 (0.0%)	1 of 2 (50.0%)

Numbers are No. (%)

## Discussion

This cohort study included 419 RSLs diagnosed on image-guided needle biopsy during an era where, at our institution, the majority of RSLs without concurrent atypia underwent imaging surveillance rather than surgical excision. This study has three main findings. First, repeat biopsies performed for additional sampling of RSLs diagnosed on ultrasound-guided core needle biopsy did not reveal any malignancies, and were unlikely to identify atypia. This finding challenges the need for additional sampling. Second, the majority of RSLs remained stable on imaging during follow-up and did not require surgical excision. And, third, when subjected to surgical excision during the initial work-up or due to a change in lesion appearance on follow-up imaging, no malignancies were diagnosed. In summary, these findings add to the evidence that RSLs without atypia are benign lesions that are very unlikely to harbor malignancy, and that imaging surveillance is a safe management strategy.

Recent studies provide additional evidence supporting the finding that RSLs without atypia have a very low likelihood of upgrade to malignancy, particularly when “adequately sampled” and radiologic-pathologic concordance is established [15, 22]. But what counts as “adequate sampling” varies and is often left unspecified. The National Comprehensive Cancer Network Breast Cancer Screening and Diagnosis guidelines recommend imaging follow-up for “adequately sampled” RSLs, and discuss the potential need for additional tissue to establish the diagnosis, which can be obtained via excisional biopsy or repeat needle biopsy [14]. The American Society of Breast Surgeons Surgical Management of Benign or High-Risk Lesions resource guide recommends surveillance for “adequately sampled” RSLs, noting that this approach is reasonable “depending on the imaging finding, lesion size, and biopsy method.” [13].

Other guidelines are more explicit about the meaning of “adequate sampling,” but favor more extensive procedures prior to observation in patients diagnosed with RSLs. European guidelines recommend that RSLs diagnosed by 14-gauge core needle biopsy or by VAB are best managed by subsequent vacuum-assisted excision for thorough sampling (> 4 g of tissue sampled), ideally removing the lesion in its entirety [23, 24]. Likewise, panelists at the Third International Consensus Conference on lesions of uncertain malignant potential in the breast recommend surgical excision or vacuum-assisted excision for RSLs diagnosed on core needle biopsy, or radiological follow-up if the lesion is diagnosed on and completely excised by VAB [25].

In our study, repeat image-guided biopsies or surgical excisions did not reveal malignancy in any patients, even for those initially diagnosed by 14-gauge core needle biopsy. Other recent studies [11, 26], including one where almost all patients underwent core needle biopsy [27], provide further support for the finding that the upgrade rate of pure RSLs is low, regardless of the biopsy technique utilized. These findings support consideration of observation without further tissue sampling in patients diagnosed using core needle biopsy, a strategy which could reduce healthcare costs, patient anxiety, and VAB-associated complications [28–30]. Importantly, embarking on this strategy requires close radiologic-pathologic concordance.

One way of establishing such concordance is by case presentation at a multidisciplinary case conference (MCC) for benign and high-risk breast lesions [31]. RSLs have historically been presented at our institution’s MCC. But, in recent years, we have discontinued presentation of RSLs diagnosed by VAB (9-gauge needle and at least 12 cores), given their extremely low likelihood of upgrade reported in prior studies from our institution [32, 33]; concordance for these lesions is assessed by the breast radiologist performing the biopsy. This present study provides evidence that VAB may not be necessary to establish the safety of observation for RSLs diagnosed by US-guided core needle biopsy. MCC presentation can be a useful avenue for clarifying concerns around radiologic-pathologic discordance or inadequate sampling prior to subjecting patients to a recommendation for an additional procedure.

Our study reports on a large number of patients who underwent imaging surveillance alone rather than surgical excision. With active surveillance becoming more widely accepted for these lesions, evidence on the safety of this strategy is needed. Other studies have reported on smaller numbers of patients undergoing surveillance alone [12, 32, 34–36], with very few or no malignancies reported during follow-up. No invasive or in situ cancers were identified at the RSL site in our study. Although our study protocol planned for subgroup analyses by lesion characteristics and patient risk factors, this was not possible due to the absence of events.

Implementing active surveillance for RSLs without atypia on the basis of the evidence presented in this study should be done with caution due to its limitations. Firstly, this study was conducted at a single tertiary cancer center, where radiologic-pathologic correlation is routinely verified and biopsy is performed by highly experienced breast radiologists. These practice patterns may limit the generalizability of our findings to centers with different biopsy techniques or less structured multidisciplinary review. In these settings, or when adherence to imaging follow-up cannot be ensured, surveillance may not be a safe strategy. Second, the median imaging follow-up of 2.7 years, while consistent with the recommended follow-up of probably benign breast lesions [14], is reflected in a wide confidence interval for the estimate of 5-year malignancy risk. While our findings strongly suggest a very low likelihood of malignant upgrade, small absolute long-term risks cannot be excluded, and continued surveillance is necessary.

## Conclusion

In this contemporary cohort of RSLs without atypia, no upgrades to DCIS or invasive carcinoma were observed at the RSL site on repeat biopsy, surgical excision, or during imaging surveillance. These findings support a management strategy of imaging surveillance, particularly when radiologic-pathologic concordance is established. Routine repeat biopsy or surgical excision for “adequate sampling” may not be necessary for most patients with pure RSLs.

## Data Availability

The datasets generated during this study are not publicly available due to institutional and patient privacy restrictions. Deidentified data may be made available from the corresponding author upon reasonable request and subject to institutional approval.
